# Intraductal and invasive adenocarcinoma of duct of Luschka, mimicking chronic cholecystitis and cholelithiasis

**DOI:** 10.1186/1477-7819-7-4

**Published:** 2009-01-07

**Authors:** Mumtaz Jahan, Philip Xiao, Alan Go, Muhammad Cheema, Arif Hameed

**Affiliations:** 1 Department of Family Practice, The Brooklyn Hospital Center, Brooklyn, NY 11201, USA; 2 Pathology and Laboratory Medicine, The Brooklyn Hospital Center, Brooklyn, NY 11201, USA; 3 General Surgery, The Brooklyn Hospital Center, Brooklyn, NY 11201, USA

## Abstract

**Background:**

Intraductal and invasive adenocarcinoma of duct of Luschka is rare. To the best of our knowledge, this is the second case report of intraductal and invasive carcinoma arising from ducts of Luschka.

**Case presentation:**

Patient presented to hospital with signs and symptoms of chronic cholecystitis and cholelithiasis. Ultrasound examination revealed thickening of gallbladder wall with abnormal septation around liver bed. Patient underwent laparoscopic cholecystectomy and resection of the adjacent liver bed. Histologic examination confirmed an intraductal and invasive adenocarcinoma arising from Luschka ducts.

**Conclusion:**

Adenocarcinoma of ducts of Luschka should be considered among differential diagnoses for the patients with typical clinical presentations of chronic cholecystitis and cholelithiasis.

## Background

Initially described by Herbert von Luschka, the ducts of Luschka are aberrant small bile duct or ductules in liver bed and/or in sub-peritoneal region around wall of gallbladder adjacent to liver bed. The incidence of duct of Luschka varies from 1 percent to 50 percent [1-3]. Florid proliferation of ducts of Luschka accompanied by cellular fibroblastic stroma and varying degree of inflammation may cause thickening of gallbladder wall around liver bed and mimic well-differentiated adenocarcinoma under microscopic examination [4]. Intraductal and invasive adenocarcinoma of duct of Luschka are rare. To the best of our knowledge, only one case has been reported [5], we here described the second case of intraductal and invasive carcinoma arising from ducts of Luschka.

## Case presentation

A 31-year year old Hispanic women presented in a family practice clinic with severe right upper quadrant pain for past several week. The abdominal pain was accompanied by nausea, vomiting and loss of appetite. Physical examination revealed tenderness in the right upper quadrant. The patient gave history of two admissions in a local ER for similar complaints where she received medical treatment. An ultrasound examination showed gallstones, thickening of gallbladder wall with abnormal septation around liver bed and congenital absence of right kidney. A pre-operative diagnosis of cholecystitis and cholelithiasis was rendered and an MRI was recommended for further evaluation of the thickened wall of gallbladder fundus in liver bed. However, the patient underwent laparoscopic cholecystectomy and resection of the adjacent liver bed. Intraoperative findings included densely adherent gallbladder in the liver bed and a thickened gallbladder fundic wall in and around cholecystic fossa. Frozen section of the thickened area was interpreted as invasive carcinoma. Patient underwent a second look exploratory laparotomy and additional resection of the liver bed. No residual intraductal or invasive adenocarcinoma was seen in the surgical specimen.

### Pathological findings

The gallbladder measures 9 × 4 × 3 cm and shows an indurated localized thickened fundic wall area measuring 2 × 1.5 × 1 cm located around the fundus and liver bed. Cut surface of the thickened area was off-white and firm. The gallbladder mucosa was normal. Three tan-yellow stones were present within gallbladder lumen. Gallbladder neck and cystic duct were grossly unremarkable.

**Figure 1:**
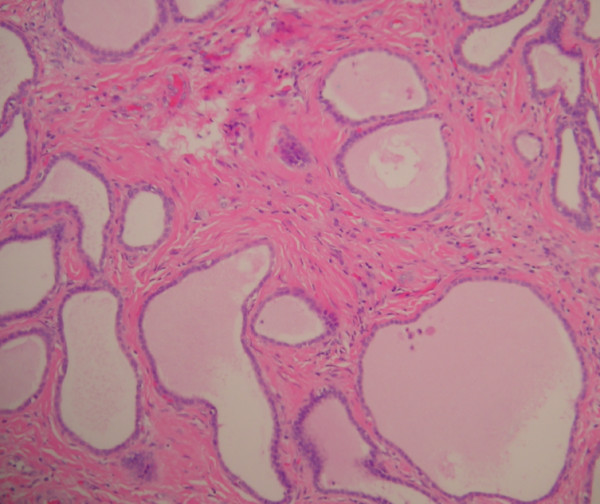
**Ducts of Luschka**.

**Figure 2:**
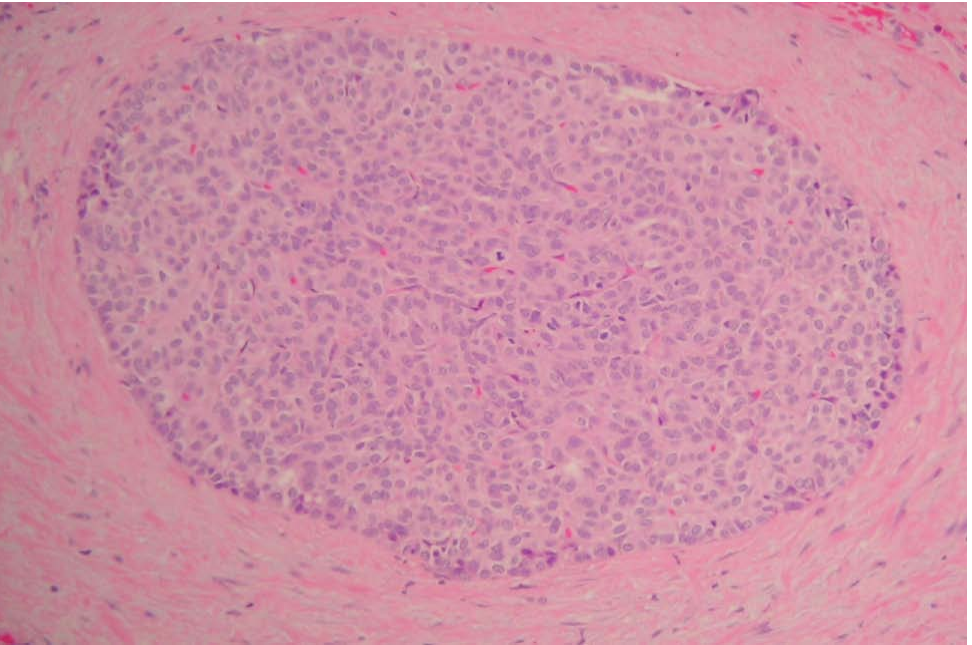
**Intraductal carcinoma of ducts of Luschka**.

**Figure 3:**
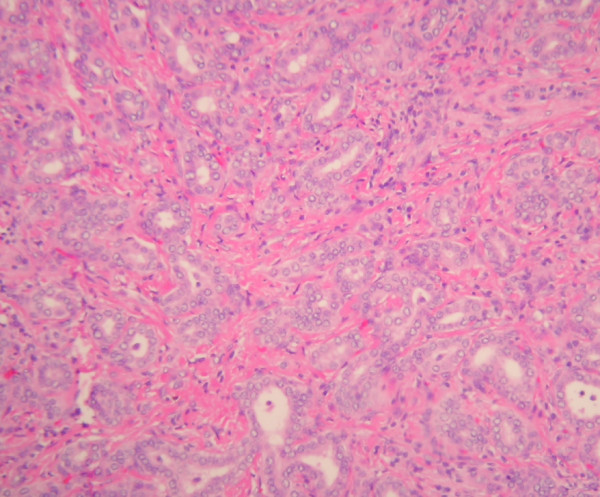
**Invasive adenocarcinoma**.

**Figure 4:**
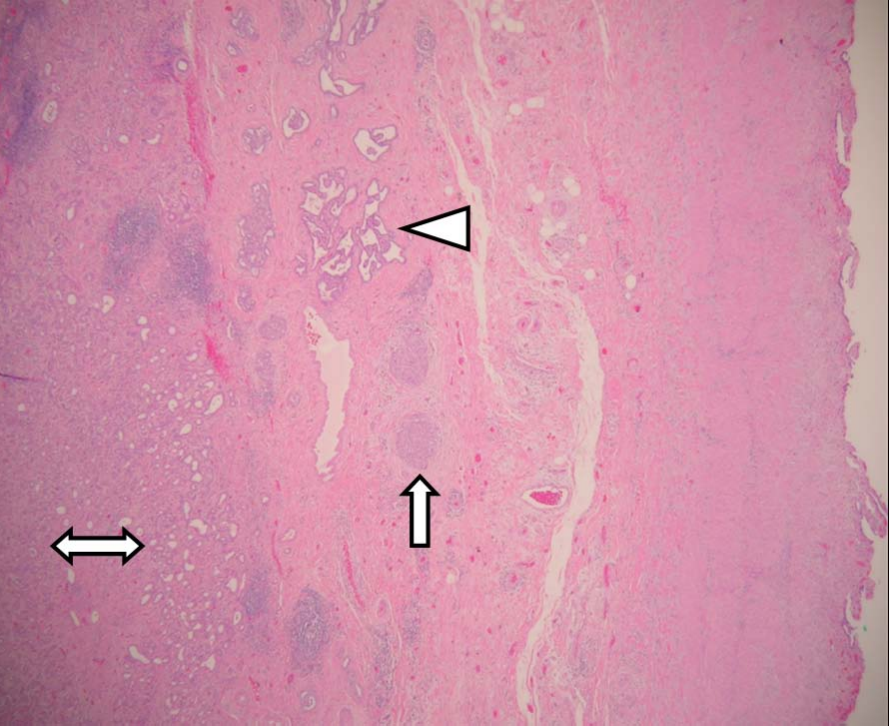
**A low power view of an area showing an invasive adenocarcinoma (double-ended arrow), intraductal carcinoma (arrow) and Luschka ducts (triangle) in sub-peritoneal connective tissue around gallbladder**.

The entire liver bed and thickened portion of gallbladder wall was submitted for histological examination. Microscopic examination showed fibrous thickening of the gallbladder bed containing a meshwork of benign ductules and small 1–2 mm thick wall ducts. Some of these ductules were cystically dilated (Figure 1). These ducts and ductules were present in liver bed and subperitoneal connective tissue around lateral fundic wall and were identified as ducts of Luschka. Some of these ductules showed intraductal epithelial hyperplasia with atypia. These ducts were intermingled with intraductal (Figure 2) and low-grade invasive ductal adenocarcinoma with desmoplastic response (Figure 3). The intraductal carcinoma involved Luschka ducts and ductules and exhibits solid epithelial growth pattern. The invasive adenocarcinoma component showed predominantly small tubular growth pattern. Foci of perineural invasion by the tumor were also seen. The intraductal and invasive ductal carcinoma involved gallbladder adventitia and extended in the sub-peritoneal region around fundic portion of the gallbladder adventitia (Figure 4). No invasion of gallbladder muscularis was seen. The tumor also minimally invaded the adjacent hepatic parenchyma in the liver bed. The gallbladder mucosa histologically was unremarkable. The surgical resection margin of the liver bed and cystic ducts were negative for the tumor. Immunohistochemical studies showed that the tumor was positive for CK7, CK19, CEA (both monoclonal/polyclonal) and negative for CK20, CDX2, TTF-1, chromogranin, synaptophysin and Estrogen/Progesterone receptors.

## Discussion

Ducts of Luschka may connect with intrahepatic bile ducts but do not communicate with gallbladder lumen. These ducts are known source of bile leak or biliary peritonitis after cholecystectomy. Anomalous bile ducts are found distributed within the connective tissue of gallbladder bed. Gallbladder in this region is not covered by serosa and connective tissue layer is continuous with interlobular connective tissue of the liver. Gallbladder bed may contain two types of anomalous bile ducts: bile duct of Luschka and cystohepatic duct [1]. Ducts of Luschka are 1–2 mm in diameter which drain sub-segment of right liver lobe into right hepatic, common hepatic and cystic ducts. Ducts of Luschka occur commonly in the center of the gallbladder bed as well as in the region of lateral subperitoneal reflection. Microscopic examination of bile ducts of Luschka shows that it is a meshwork of tiny ductules rather than a single duct. Bile ducts of Luschka reach the adventitial layer but do not enter the lumen of the gallbladder. The cystohepatic duct is a thick wall duct that courses through gallbladder fossa or in the posterior gallbladder wall and typically enters into cystic duct or right hepatic duct. Prevalence of cystohepatic duct has been estimated at 1–2% of the surgical cases. Like duct of Luschka, cholecystectomy may result in inadvertent injury to a cystohepatic duct and bile leakage [6,7].

Gross thickening of fundic portion of gallbladder wall is seen in such benign lesions as xanthomatous cholecystitis, adenomyomatous hyperplasia, Rokitansky-Aschoff sinuses and florid proliferation of bile ducts of Luschka. Likewise gallbladder adenocarcinoma may produce similar gross thickening of fundic portion of the gallbladder. Therefore above described entities constitute important differential diagnoses of lesions causing thickening of gallbladder wall.

The adenocarcinoma described in this report seems to be arising from Luschka ducts. The following observations support its origin from the duct of Luschka: 1) The bulk of the tumor is confined to the gallbladder bed between liver, fundic portion of gallbladder wall, and lateral subperitoneal connective tissue with minimal invasion into the adjacent liver and adventitial layer of gallbladder. 2) The coexistence of foci of intraductal carcinoma within some of associated Luschka ducts/ductules. 3). The co-existence of benign small isolated thick wall ducts of 1–2 mm diameter as well as meshwork of Luschka ductules in gallbladder bed adjacent to the tumor.4) Absence of a primary tumor elsewhere. 5) The presence of the tumor in an area where duct of Luschka are normally prevalent.

It may be difficult to delineate exact origin of a locally advanced malignancy from biliary structures such as gallbladder wall, Luschka ducts, cystohepatic ducts and liver within gallbladder fossa because of their close proximity. One can speculate that some of the locally advanced bulky tumors in cholecystic fossa [8] could potentially have their origin from Luschka duct. The tumor described here was relatively small with preservation of normal anatomical landmark including Luschka duct and minimal invasion into liver and gallbladder adventitia which facilitated identification of its precise origin. Adenocarcinoma of ducts of Luschka should be considered among differential diagnoses for the patients with typical clinical presentations of chronic cholecystitis and cholelithiasis.

## Consent

Written consent was obtained from the patient for publication of this case report, a copy of this consent is available with editorial office.

## Competing interests

The authors declare that they have no competing interests.

## Authors' contributions

MJ wrote the case history and collected all clinical information. PX was responsible for literature review, medline search and wrote the first draft. AG reviewed the paper and made suggestions. MC reviewed the paper and made suggestions. AH interpreted data and critically revised the manuscript. All authors read and approved the final manuscript.
